# Traumaassoziierte Gefäßverletzungen und deren gefäßchirurgische/interventionelle Rekonstruktionsmöglichkeiten

**DOI:** 10.1007/s00104-024-02124-8

**Published:** 2024-07-22

**Authors:** U. Barth, S. Piatek, M. Stojkova, H. Krause, F. Meyer, Z. Halloul

**Affiliations:** 1 Arbeitsbereich Gefäßchirurgie, Klinik für Allgemein‑, Viszeral‑, Gefäß- & Transplantationschirurgie, Universitätsklinikum Magdeburg A. ö. R., 39120 Magdeburg, Deutschland; 2 Universitätsklinik für Unfallchirurgie, Universitätsklinikum Magdeburg A. ö. R., Magdeburg, Deutschland; 3 Universitätsklinik für Radiologie und Nuklearmedizin, Universitätsklinikum Magdeburg A. ö. R., Magdeburg, Deutschland; 4 Abteilung für Kinderchirurgie, Kindertraumatologie und Kinderurologie, Klinik für Allgemein‑, Viszeral‑, Gefäß- & Transplantationschirurgie, Universitätsklinikum Magdeburg A. ö. R., Magdeburg, Deutschland; 5 Klinik für Allgemein‑, Viszeral‑, Gefäß- & Transplantationschirurgie, Universitätsklinikum Magdeburg A. ö. R., Magdeburg, Deutschland

**Keywords:** Traumatisches Pseudoaneurysma, Traumatische Aortenruptur, Suprakondyläre Humerusfraktur, Endovaskuläre Techniken, Traumatic pseudoaneurysm, Traumatic aortic rupture, Supracondylar humeral fracture, Endovascular techniques

## Abstract

**Ziel:**

Ziel der Arbeit ist die Darstellung der Diversität von Gefäßverletzungen in Bezug auf Gefäßsegmente bzw. Körperregionen, Unfallmechanismen und spezifische Patientenkonstellationen.

**Methode:**

Es erfolgte eine repräsentative Fallsammlung(-serie).

**Ergebnisse:**

Die Diagnostik von Gefäßverletzungen im Rahmen von Traumata und Frakturen beginnt mit einer gründlichen körperlichen Untersuchung. Zusätzlich sollten die von der Western Trauma Association präferierten harten und weichen Zeichen in die Entscheidung mit einbezogen werden. Die Ultraschall-Doppler-Untersuchung ist durch vergleichende Messungen das sicherste und schonendste nichtinvasive Untersuchungsverfahren beim Verdacht auf eine Gefäßverletzung. Die Stabilisierung einer Fraktur, ideal mittels Fixateur externe, sollte möglichst vor der Gefäßrekonstruktion erfolgen, es sei denn, dass massive Blutung, hypovolämischer Schock oder ein sich rasch ausbreitendes Hämatom eine sofortige Indikation zum Eingriff darstellen. Bei kindlichen suprakondylären Frakturen wird häufig eine Gefäßverletzung ohne relevante Ischämie beschrieben („pink pulseless hand“). Hier sollte zunächst die Reposition der Fraktur erfolgen, weil es häufig wieder zu einer Erholung des Pulses kommt. Aufgrund der zunehmenden Verfügbarkeit, der guten technischen Handhabung und der hohen technischen Erfolgsquote sowie des eher limitierten Interventionstraumas hat sich eine endovaskuläre Versorgung traumatisch bedingter Gefäßverletzungen weitgehend durchgesetzt. Traumatische Aortenrupturen sind mit einer hohen Mortalität bereits am Unfallort behaftet. Die schnelle endovaskuläre Versorgung mittels einer Stentprothese erhöht die Überlebenschancen des Verletzten deutlich.

**Schlussfolgerung:**

Gefäßverletzungen im Zusammenhang mit Frakturen oder Mehrfachverletzungen erfordern ein interdisziplinäres Zusammenspiel der beteiligten Fachgebiete.

## Abstract (en)

**Aim:**

The aim of this work is to illustrate the diversity of vascular injuries in terms of vascular segments or body regions, accident mechanisms and specific patient constellations.

**Method:**

A representative case collection was compiled based on current and relevant scientific references in PubMed, own clinical experiences, vascular surgical and novel image-guided interventional options.

**Results:**

The diagnostics of vascular injuries in the context of trauma and fractures are based on a thorough physical examination. In addition, the hard and soft signs preferred by the Western Trauma Association should be included in the decision. Doppler ultrasonography examination is the safest and gentlest noninvasive examination procedure for a suspected vascular injury due to repeatable and comparative measurements. The stabilization of a fracture, ideally using an external fixator, should be performed before vascular reconstruction whenever possible, unless massive bleeding, hypovolemic shock or a rapidly spreading hematoma represent an immediate indication for surgery. In pediatric supracondylar fractures, avascular injury without relevant ischemia has frequently been described (pink pulseless hand). In this case, the fracture should first be reduced as the pulse often recovers. Due to the increasing availability, good technical handling and high technical success rate as well as the relatively limited interventional trauma, endovascular treatment of traumatic vascular injuries has become widely accepted. Traumatic aortic ruptures are associated with a high mortality even at the accident site. Rapid endovascular treatment using a stent prosthesis significantly increases the injured person’s chances of survival.

**Conclusion:**

Vascular injuries in connection with fractures or multiple injuries require interdisciplinary cooperation between the specialties involved.

## Hintergrund

Nahezu täglich stellen sich Kinder und Erwachsene in den Notaufnahmen mit Frakturen vor.

Äußerst selten kommt es im Verhältnis hierbei zu Verletzungen der arteriellen Gefäße mit Ausbildung entsprechender Blutungskomplikationen oder Bildung von Pseudoaneurysmen. Die Inzidenz einer relevanten Gefäßverletzung im Rahmen eines stumpfen Traumas liegt bei unter 4 % und bei penetrierendem Trauma bei unter 30 %. Mehrfach Schwerverletzte weisen in bis zu 10 % der Fälle eine relevante Gefäßbeteiligung auf. Die Mortalitätsrate liegt zwischen 2–12 % [1].

Verletzungen z. B. der A. subclavia und A. axillaris sind mit einer hohen Morbidität und Mortalität verbunden, die nach Literaturangaben bei 15–34 % liegen können [2]. Dies musste auch vor mehr als 5000 Jahren der Steinzeitmensch „Ötzi“ erleben, dessen Pfeilschusswunde nach radiologischer Rekonstruktion wahrscheinlich durch Entstehung eines Pseudoaneurysmas der linken A. subclavia zum Tode führte [3]. Im Bereich der unteren Extremitäten betreffen 60 % der peripheren Gefäßverletzungen die femoropoplitealen Gefäße [4]. Drohende Folgen sind ein lebensbedrohlicher Blutverlust und ein hohes Amputationsrisiko. Im eigentlichen Sinne sind die Gefäß-Nerven-Bündel durch die begleitenden Knochenstrukturen geschützt, sodass nur schwere Traumen mit Dislokation der Frakturenden und Verletzung der Gefäßwand zu Blutungen und Ausbildung von Pseudoaneurysmen führen können. Trotz der Verbesserung der Sicherungssysteme in Fahrzeugen kommt es immer wieder zu schweren Verkehrsunfällen mit komplizierten Verletzungen, die fachübergreifend die behandelnden Ärzte vor Herausforderungen stellen.

In den letzten Jahren hat sich das Portfolio der therapeutischen Möglichkeiten bei begleitenden Gefäßverletzungen durch die Entwicklung der endovaskulären gefäßchirurgischen/interventionell-radiologischen Therapien deutlich erweitert. Deshalb ist die Präsentation der Diagnostik und Therapie mehrerer interessanter und repräsentativer Fälle mit Darstellung der modernen therapeutischen Möglichkeiten von wissenschaftlichem Interesse und dient einer aktualisierten Wissensvermittlung.

## Material und Methoden

Die klinische Fallserie (Design) mit Diskussion soll auf Basis eigener operativer und klinischer Erfahrungen, insbesondere untersetzt mit eigenen Fallzahlen von Gefäßverletzungen bei kindlichen Frakturen und traumatischen Aortenverletzungen, sowie aktueller und einschlägiger wissenschaftlicher Referenzen in PubMed,

- das entscheidungstechnische Herangehen,
- das taktische Vorgehen,
- die operativen Möglichkeiten (sowie)
- deren perioperatives Management

anhand 6 ausgewählter Fallkomplexe diverser

- Gefäßsegmente bzw. Körperregionen,
- Unfallmechanismen (bzw.)
- Patientenkonstellationen (Alter, Zeitgang)

darstellen.

## Kasuistiken

### Fallbericht 1

Eine 57-jährige Patientin erlitt 4 Monate vor der gefäßchirurgischen Vorstellung ein Sturztrauma auf die linke Schulter. Im Verlauf kam es zur Entwicklung eines pulsierenden Tumors des linken klavikulären Bereiches mit Ausbildung eines zunehmenden Stauungsödems des linken Arms, venöser subkutaner Umgehungskreisläufe und einer Dermatitis des linken Armes.

**Abb. 1:**
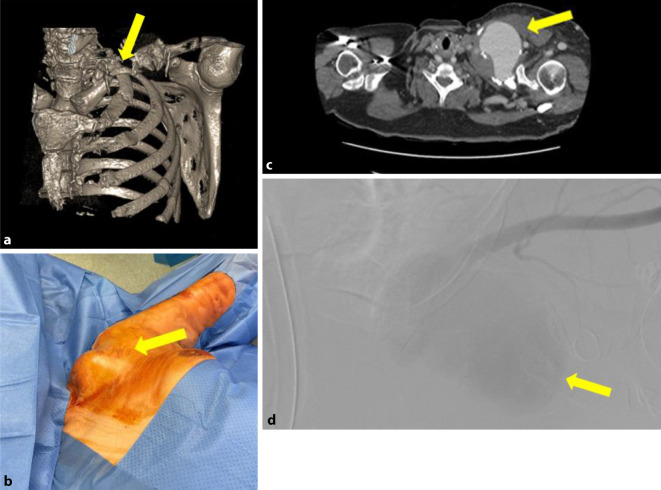
Fall 1: Pseudoaneurysma der A. subclavia links. **a** 3-D-Rekonstruktion des linksthorakalen CT-Scans, insbesondere der linken Klavikula mit Osteolyse im mittleren Drittel (*gelber Pfeil*), **b** präoperative Darstellung des linksklavikulären Lokalbefundes mit raumfordernder Wirkung (*gelber Pfeil*), **c** CT-Angiographie (transversale Schichtung) mit Nachweis des Pseudoaneurysmas links (*gelber Pfeil*), **d** digitale Subtraktionsangiographie der A. subclavia sinistra mit Nachweis des Pseudoaneurysmas (*mattgrau* – *gelber Pfeil*)

Die initiale Sonographie zeigte eine 7,1 × 7,2 cm echoleere Raumforderung mit echoreichen Thrombusformationen, in der farbcodierten Duplexsonographie mit einer pulssynchronen vollen Farbfüllung. Die konventionelle Nativröntgenuntersuchung der linken Schulterregion (und) die Knochenrekonstruktion einer Nativcomputertomographie (CT) dokumentierten eine ausgedehnte Osteolyse der Klavikula im mittleren Drittel, die eine Längsausdehnung von ca. 5–6 cm im Vergleich zur Gegenseite einnahm (Abb. 1a). Es ließ sich eine ausgedehnte Weichteilschwellung mit einem Durchmesser von 103 mm in dieser Region nachweisen (Abb. 1b). Die nachgeschaltete CT-Angiographie (CTA) zeigte im Bereich der linken Schulter ein ca. 8,6 × 7 cm großes Pseudoaneurysma, entspringend aus der A. subclavia sinistra, welches die Umgebungsstrukturen verdrängte (Abb. 1c).

Eine komplettierende digitale Subtraktionsangiographie (DSA) identifizierte das Pseudoaneurysma, entspringend aus einem Defekt der A. subclavia sinistra (Abb. 1d).

Aufgrund der ausgeprägten Umgebungsreaktion wurde die Indikation zur offenen arteriellen Rekonstruktion im A.-subclavia-Stromgebiet links mit infraklavikulärem Zugang gestellt.

Bei der Operation erfolgte die Freilegung des durch eine kräftige Kapsel geschützten Pseudoaneurysmas, Eröffnung desselben und Entfernung von Hämatom und Thrombussaum. Die Kapsel wurde zu zwei Drittel reseziert und nach Darstellung der Perforationsstelle erfolgte der Direktverschluss mittels Übernähung durch eine fortlaufende Naht mit nichtresorbierbarem Nahtmaterial 4/0 (Prolene, Ethicon, Johnson & Johnson Medical, New Brunswick/NJ, USA). Des Weiteren erfolgte die Entfernung des ursächlichen spitzen Knochenendes der Klavikula. Histologisch zeigte sich eine Geflechtknochenbildung und eine fokal osteoklastische Reaktion, Blutungsresiduen und eine ausgeprägte narbige Fibrose in der Umgebung einschließlich granulierender Reaktion ohne Anhalt für Malignität. Diese Veränderungen wurden im Rahmen der zellulären Reaktionen im Sinne einer avitalen Pseudoarthrosebildung gesehen. Eine funktionelle Einschränkung durch den Defekt wurde von der Patientin verneint, sodass bei Abwägung des Risiko-Nutzen-Verhältnisses von einer aufwendigen Rekonstruktion mit Anlagerung einer autologen Spongiosa oder eines kortikospongiösen Spans und Plattenrekonstruktion Abstand genommen wurde. In den postoperativen Kontrollen war kein neurologisches Defizit im Bereich des linken Armes als Ausdruck einer Plexusschädigung festzustellen. Eine normale Perfusion des linken Armes und ein deutlicher Rückgang der Schwellung bestätigten ein regelrechtes postoperatives Ergebnis.

### Fallbericht 2

**Abb. 2:**
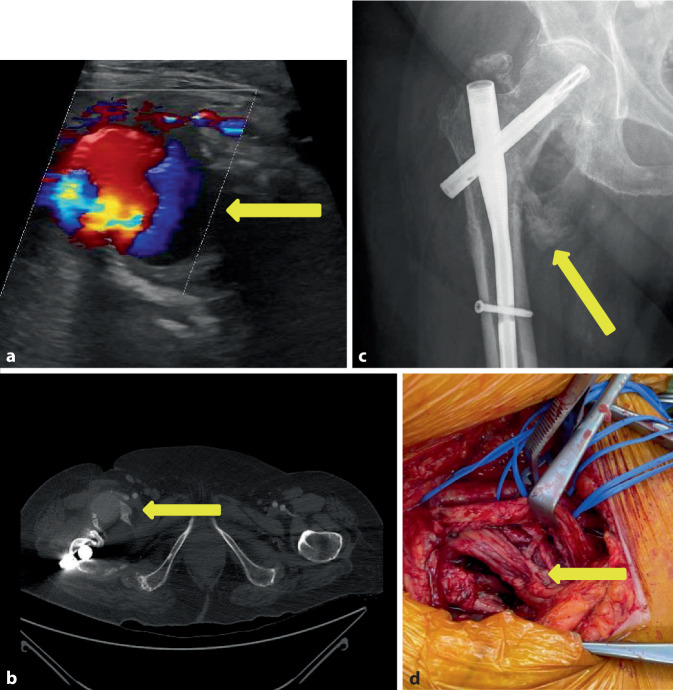
Fall 2: Pseudoaneurysma der A. profunda femoris rechts.** a** Farbkodierte Duplexsonographie des Pseudoaneurysmas mit typischem Farbwechsel (*gelber Pfeil*),** b** CT-Angiographie mit Nachweis des Pseudoaneurysmas der rechten Inguinalregion (*gelber Pfeil*),** c** Nativröntgen der mit einem PFN-Nagel versorgten pertrochantären Fraktur mit zirkulärer Verkalkung (*gelber Pfeil*),** d** intraoperative Darstellung der Pseudoaneurysmahöhle und des verletzten Profundaastes (*gelber Pfeil*)

Im Rahmen eines Sturzes erlitt eine 92-jährige Patientin eine pertrochantäre Femurfraktur rechtsseitig, die durch eine geschlossene Reposition und Osteosynthese mittels proximalen Femurnagels Antirotation (PFNA)-Verriegelungsnagel (Fa. DePuy Synthes, Oberdorf, Schweiz) rechts versorgt wurde. Bei guter physischer und psychischer Compliance wurde die Patientin postoperativ frühzeitig mobilisiert und in die Häuslichkeit entlassen. Die Vorstellung in der Gefäßchirurgie erfolgte nach 2 Monaten aufgrund einer peripheren arteriellen Verschlusskrankheit (pAVK) Stadium IV mit Zehennekrosen rechts. Die Diagnostik mittels farbkodierter Duplexsonographie zeigte eine ca. faustgroße echoleere Raumforderung neben dem Schenkelhals mit einer pulssynchronen Verwirbelung im Sinne eines Pseudoaneurysmas (Abb. 2a). Die CTA ergab neben einer ausgeprägten Unterschenkel-pAVK rechts ein monströses Pseudoaneurysma der A. profunda femoris rechts mit ausgeprägter perifokaler Verkalkung (Abb. 2b). Bereits in einer Nativröntgenaufnahme ist medial des Schenkelhalses eine schalenförmige Verkalkungsstruktur zu sehen (Abb. 2c). Aufgrund der begleitenden kritischen Ischämie des rechten Unterschenkels erfolgte der kombinierte Eingriff der offenen Resektion des Pseudoaneurysmas und Ligatur des perforierten Profundaastes (Abb. 2d) sowie die begleitende Ballonangioplastie des Unterschenkels, worunter es zu einer Stabilisierung der pAVK Stadium IV rechtsseitig kam. Der genaue Mechanismus der Entwicklung des Pseudoaneurysmas verblieb letztlich nicht völlig geklärt, da der Operationsbericht keinen Hinweis auf einen untypischen Operationsverlauf ergab.

### Fallbericht 3

**Abb. 3:**
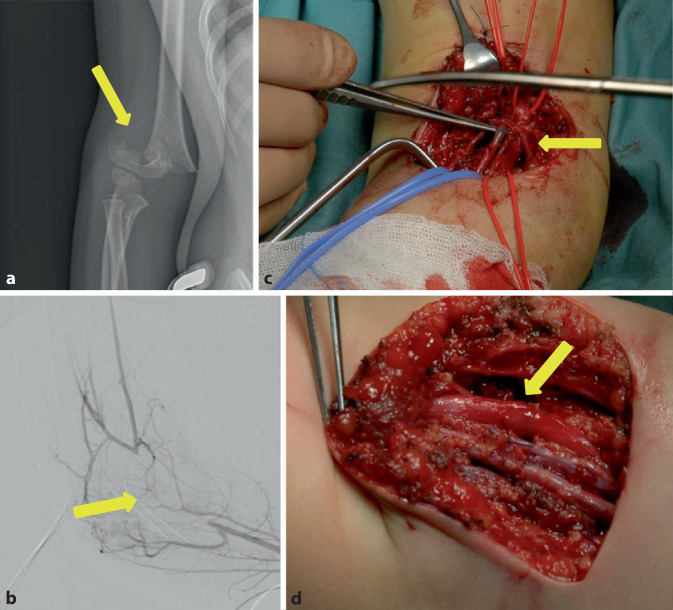
Fall 3: Thrombose der A. brachialis nach suprakondylärer Humerusfraktur rechts. **a** Röntgennativbild der suprakondylären Humerusfraktur rechts (anterior/posterior, *gelber Pfeil*), **b** digitale Subtraktionsangiographie des rechten Armes mit Nachweis des thrombotischen Verschlusses der A. brachialis rechts (*gelber Pfeil*), **c** intraoperatives Bild der thrombosierten A. brachialis rechts (*gelber Pfeil*), **d** intraoperatives Bild der rekonstruierten A. brachialis rechts (*gelber Pfeil*)

Das dreieinhalbjährige Mädchen hatte sich beim Trampolinspringen eine suprakondyläre Humerusfraktur (Abb. 3a) rechts zugezogen. Nach Erstversorgung in einem auswärtigen Krankenhaus und temporärer Ruhigstellung in einer Armschiene erfolgte die Verlegung des Kindes in die Kinderchirurgie der hiesigen Klinik. Bei der Erstuntersuchung war die Sensibilität bei ängstlichem Verhalten des Kindes nur eingeschränkt beurteilbar, die Motorik schmerzbedingt eingeschränkt. Die Pulse an der A. radialis und ulnaris waren im Vergleich zur Gegenseite abgeschwächt, das kapilläre „Refill“ jedoch erhalten. Es erfolgte zunächst die operative Reposition und Stabilisierung mittels K‑Draht-Osteosynthese. Postoperativ wurde die rechte Hand konsequent pulsoxymetrisch überwacht. Bei einer 100 %igen Sättigung zeigten sich regelhafte Pulskurven. Eine farbkodierte Duplexsonographie der A. radialis und ulnaris zeigte pathologische Flusskurven, sodass anschließend eine DSA durchgeführt wurde (Abb. 3b), die einen Komplettverschluss der A. cubitalis rechts zeigte, der durch Kollateralisierung überbrückt wurde. Daraufhin wurde die Indikation zur operativen Freilegung gestellt. Hier zeigte sich die A. brachialis in den Frakturspalt eingezogen mit einem thrombotischen Verschluss (Abb. 3c). Nach Arteriotomie erfolgte die Thrombektomie und die Patchplastik mit einem Transplantat aus der V. mediana cubiti rechts (Abb. 3d). Nach Beendigung der Patchplastik und Freigabe des Blutstroms konnte ein regelrechtes Doppler-Signal mit der Mikro-Doppler-Sonde abgeleitet werden. Der weitere postoperative Verlauf gestaltete sich komplikationslos bei regelrechter Pulsation von A. radialis und A. ulnaris im Verlauf.

### Fallbericht 4

**Abb. 4:**
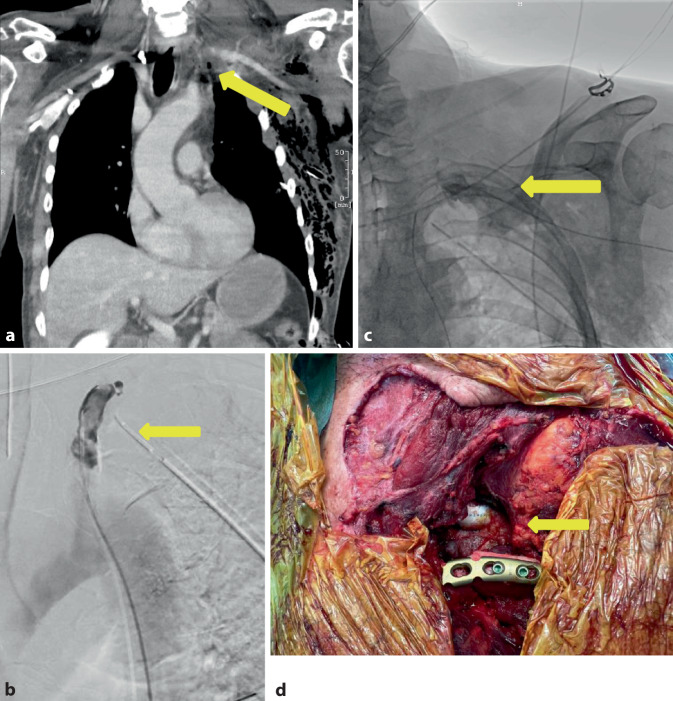
Fall 4: Traumatische Verletzung der A. subclavia links. **a** CT-Angiographie mit Nachweis des Verschlusses der A. subclavia links (*gelber Pfeil*), **b** Angiographie mit Versuch der Sondierung des Verschlusses vom Abgang aus der Aorta (*gelber Pfeil*), **c** Angiographie mit Sondierung des Verschlusses im Rendesvouz-Verfahren zusätzlich von transbrachial (*gelber Pfeil*), **d** Operationssitus mit sichtbarer PTFE-Prothese und rekonstruierter Klavikula mit Platte (*gelber Pfeil*)

Der 60-jährige Patient fuhr mit seinem PKW in den Abendstunden ungebremst auf ein stehendes Fahrzeug auf und zog sich eine penetrierende Verletzung der A. subclavia links im Bereich des Abganges der A. vertebralis links mit akuter Ischämie des linken Armes, einem großen Haut-Weichteil-Defekt im Bereich des linken unteren Halsdreiecks mit Abriss des M. sternocleidomastoideus, eine drittgradig offene Klavikulafraktur links mit ausgeprägtem supra- und infraklavikulärem Hämatom, ein Emphysem und einen Hämatopneumothorax links bei Rippenserienfraktur links und eine offene Unterkieferfraktur links zu. Die CTA zeigte kurz hinter dem Abgang aus der Aorta einen Verschluss der A. subclavia links über eine Länge von ca. 5 cm im Bereich der bestehenden Klavikulafraktur (Abb. 4a). Bei stabilen Kreislaufverhältnissen entschied man sich zum Versuch der interventionellen endovaskulären Rekanalisation des Subklaviaverschlusses (Abb. 4b, c). Dieser ließ sich jedoch nicht sondieren und versorgen, sodass bei fortgeschrittener Ischämiezeit des Armes zeitnah die Indikation zur offenen Revaskularisation gestellt wurde. Intraoperativ zeigte sich das Bild einer langstreckig mazerierten A. subclavia mit langstreckiger intravasaler Dissektion und multiplen thrombosierten Intimadefekten, die in den Abgangsbereich der A. vertebralis, A. mammaria, Truncus thyreocervicalis und Truncus costocervicalis reichte. Es wurde die aufwendige Intimarekonstruktion und nachfolgende Patchplastik mit einem XenoSure® Patch (Le Maitre Vascular, Burlington/VT, USA) zum Erhalt der Gefäßabgänge durchgeführt. Bei sich weiter fortsetzender Dissektion nach zentral mit thrombotischem Verschluss erfolgte nach zentral die Ligatur der A. subclavia und Anlage eines karotidosubklavialen Bypasses mit einer beringten Polytetrafluorethylen(PTFE)-Prothese (Gore Propaten®, 8 mm, W. L. Gore & Associates, Inc., Putzbrunn, Deutschland) mit distalem Anschluss auf die Patchplastik (Abb. 4d). Im Anschluss erfolgte die Versorgung der Klavikulafraktur mit einer winkelstabilen Platte. Aufgrund der überlangen Ischämiezeit erfolgte die prophylaktische Kompartmentspaltung am Arm. Die Aa. radialis und ulnaris waren klinisch unmittelbar nach Rekonstruktion kräftig tastbar und mit dem „Hand-Doppler“ ableitbar.

### Fallbericht 5

**Abb. 5:**
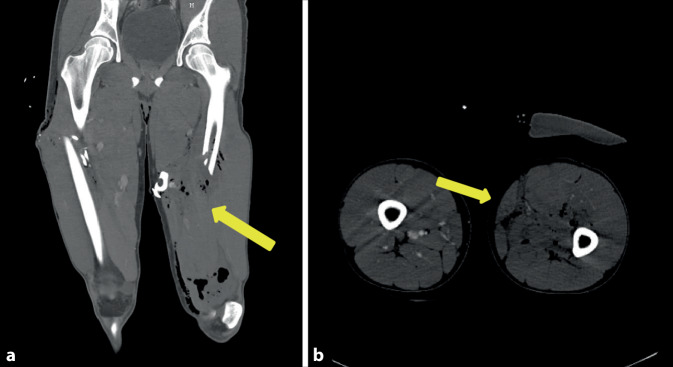
Fall 5: Traumatische Verletzung der A. und V. femoralis superficialis bei drittgradig offener Femurfraktur links. **a** CT-Angiographie in koronarer Schichtung mit Darstellung der Oberschenkelfraktur und Lufteinschlüssen (*gelber Pfeil*), **b** CT-Angiographie in axialer Schichtung mit fehlender Kontrastmittelfüllung der V. und A. femoralis superficialis links (*gelber Pfeil*)

Bei einem Verkehrsunfall zog sich der 17-jährige Patient im Rahmen eines Polytraumas eine drittgradig offene Femurfraktur linksseitig mit Verletzung der A. und V. femoralis (Abb. 5a, b) mit ausgeprägtem Haut- und Weichteilschaden zu. Zudem bestand eine Verletzung des N. femoralis links, eine erstgradig offene Femurfraktur rechts, eine Lungenkontusion beidseits sowie ein hämorrhagischer Schock. Nach Stabilisierung mittels Fixateur externe erfolgte die Revaskularisation mittels extraanatomischem femoropoplitealen Venenbypass in „Reversed“-Technik auf das P3-Segment der A. poplitea linksseitig. Als Bypassmaterial wurde die ipsilaterale V. saphena magna verwendet, die in gesamter Länge als Bypassmaterial geeignet war. Die verletzte V. femoralis superficialis wurde nicht rekonstruiert und mit Umstechungsligaturen versorgt. Unmittelbar postoperativ war eine regelrechte kapilläre arterielle und venöse Füllung im Bereich des linken Fußes zu verzeichnen.

### Fallbericht 6

**Abb. 6:**
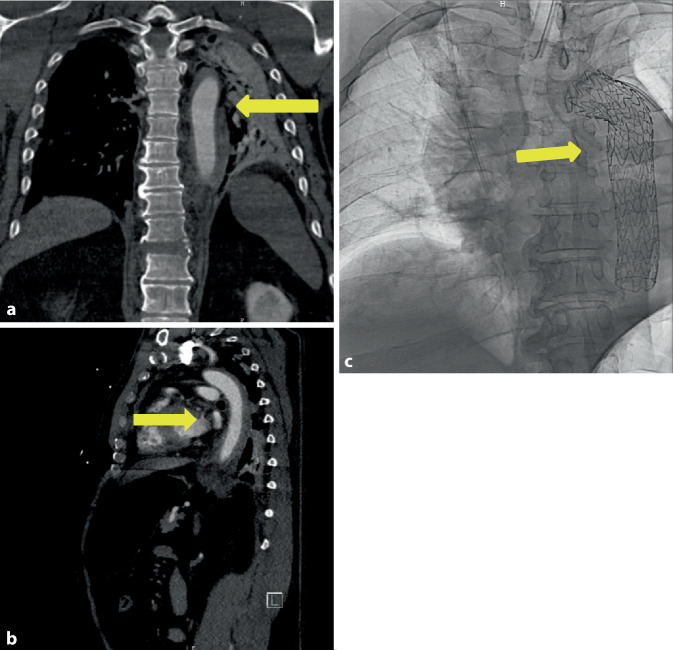
Fall 6: Traumatische Dissektion/Hämatom der Aorta thoracica nach Verkehrsunfall. **a** CT-Angiographie mit koronarer Darstellung des Aortenwandhämatoms (*gelber Pfeil*), **b** CT-Angiographie mit sagittaler Darstellung des Aortenwandhämatoms (*gelber Pfeil*), **c** Nativröntgenbild des Aortenstents (*gelber Pfeil*)

Der 58-jährige Patient fuhr mit seinem LKW gegen ein Hindernis und musste von der Feuerwehr geborgen werden. Im Rahmen des Unfalls zog er sich folgende Verletzungen zu: eine gering dislozierte Fraktur der Okzipitalkondyle links, eine kleine Kontusionsblutung temporal und basofrontal links, eine diskrete Subarachnoidalblutung hochparietal rechts, eine Rippenserienfraktur rechtsdorsal, eine Sternumfraktur, eine instabile Zerreißungsfraktur von Brustwirbelkörper (BWK) 10 mit Beteiligung des Bandscheibenfaches BWK 10/BWK 11, eine Kompressionsfraktur des Lendenwirbelkörpers (LWK) 1, eine mehrfragmentäre Fraktur des Azetabulums links, eine symphysennahe Fraktur des unteren Schambeinastes sowie eine traumatische Aortendissektion an *loco typico* des Aortenisthmus. Nach Sicherung der Aortendissektion in der Traumaspirale und einer CT-Angiographie (Abb. 6a, b) des Schockraumes erfolgte die zeitnahe Versorgung der Dissektion in der Angio-Suite mittels einer Stentprothese (Gore TAG Conformable® 28 × 28 × 150 mm, W. L. Gore & Associates, Inc.) über einen Leistenzugang rechtsseitig, beginnend am Abgang der A. subclavia links. Somit wurde das wahrscheinliche „Entry“ an typischer Stelle abgedeckt. Postoperativ blieb der Patient weiter stabil intubiert und beatmet, sodass die weitere Versorgung der Verletzungsfolgen vorgenommen werden konnte.

## Diskussion

Gefäßverletzungen werden nach der Art der Gewalteinwirkung in direkte (95 %) und indirekte (5 %) Verletzungen unterschieden. Während direkte Gefäßverletzungen durch unmittelbare Gewalteinwirkung auf das Gefäß entstehen, werden indirekte Gefäßverletzungen durch die Übertragung der äußeren Gewalteinwirkung entfernt von der Verletzungslokalisation über die umgebenden Strukturen auf die Gefäße übertragen [5]. Direkte scharfe Gefäßverletzungen werden nach Lindner und Vollmar (1965) in 3 Grade eingeteilt:

- Grad I: scharfe Durchtrennung der Adventitia mit oder ohne Media ohne Eröffnung des Gefäßlumens,
- Grad II: Eröffnung des Gefäßlumens ohne Gefäßdurchtrennung,
- Grad III: komplette Gefäßdurchtrennung (Transsektion; [5]).

Stumpfe Gefäßverletzungen werden nach Sencert (1918) ebenfalls in 3 Schweregrade eingeteilt:

- Grad I: Gefäßquetschung mit einem Intimaeinriss ohne initiale Blutung oder Ischämie. Sekundär kann ein thrombotischer Gefäßverschluss auftreten.
- Grad II: Es besteht eine Intimaschädigung mit arterieller Thrombose. Spätfolge kann ein posttraumatisches Aneurysma sein.
- Grad III: Es besteht eine vollständige Zerstörung des Arterienwandaufbaus mit Gefäßverschluss. Eine taillenartige Gefäßeinengung ist typisch, sie wird als Sanduhrphänomen beschrieben [6].

### Diagnostik

Die aufgeführten Beispiele verdeutlichen die Mannigfaltigkeit der Kombination der Verletzungsmuster mit den begleitenden Gefäßverletzungen, die die Behandler vor enorme Herausforderungen stellen können. Da sich das Behandlungsspektrum in den letzten Jahren durch den zunehmenden Einfluss der interventionellen Radiologie bzw. endovaskulären Gefäßchirurgie deutlich erweitert hat, können für den Verletzten nunmehr individualisierte Therapieoptionen genutzt werden. Trotzdem behalten die Grundregeln der Erstuntersuchung und Diagnostik weiterhin ihre Gültigkeit, um zielgerichtet Gefäßverletzungen bei Traumata zu detektieren.

Eine gründliche Untersuchung des Patienten ist in jeder Hinsicht als erster diagnostischer Schritt essenziell. Typische Hinweise für eine Gefäßverletzung sind:

- Prellmarken,
- Hautverletzungen durch penetrierende Fremdkörper,
- schmerzbedingte Funktionseinschränkungen,
- pathologische Skelettbeweglichkeit,
- örtliche oder allgemeine Blutungszeichen,
- Hautfarbe (Blässe oder livide Verfärbung),
- Temperaturdifferenzen,
- sensible und motorische Ausfälle,
- Pulsdefizit peripher,
- Dyspnoe und Dysphagie,
- Blutdruckdifferenzen [2].

Als zusätzliche Entscheidungskriterien für begleitende Gefäßverletzungen können die von der „Western Trauma Association“ präferierten harten und weichen Zeichen mit einbezogen werden (Tab. 1; [4]).

**Tab. 1:** Von der Western Trauma Association präferierte harte und weiche Zeichen [4]

Harte Zeichen	Weiche Zeichen
Pulslosigkeit	Anamnese einer starken Blutung
Blässe	Proximales Trauma
Sensibilitätsverlust	Nervale Verletzung im Verbund eines Gefäß-Nerven-Bündels
Motorikverlust	Hämatom im Verlauf einer Arterie
Schmerz	
Schnell expandierendes Hämatom	
Starke Blutung	
Tastbares oder auskultierbares Schwirren	

Die Ultraschall-Doppler-Untersuchung ist durch vergleichende Messungen das sicherste und schonendste nichtinvasive Untersuchungsverfahren beim Verdacht auf eine Gefäßverletzung [7]. Gerade bei kindlichen Verletzungen mit erschwerten Untersuchungsbedingungen durch Angst und kleinsten Bewegungsschmerz bietet sich – wie im geschilderten Fall – eine Doppler-Untersuchung mit Aufzeichnung der Flusskurven zur Detektion und Verlaufskontrolle idealerweise an. Eine DSA oder CTA ergänzt und sichert den Verdacht auf eine Gefäßbeteiligung. Bei Mehrfachverletzten oder Erwachsenen sollte die Diagnostik zeitnah durch eine CTA möglichst im Rahmen einer sog. „Traumaspirale“ komplettiert werden. Diese sollte in einer arteriellen und einer venösen Phase gefahren werden, um venöse Verletzungen miterfassen zu können. Insbesondere für die Detektion stumpfer Gefäßverletzungen kann auch eine Duplexsonographie sinnvoll sein [6], sollte aber bei schwierigen Untersuchungsbedingungen den „work flow“ nicht aufhalten. Zur primären Sicherung einer Fraktur kommt in erster Linie eine Nativröntgenaufnahme zur Anwendung. Bei klinischem Verdacht eines Pseudoaneurysmas sollte sich eine leicht durchführbare Sonographie und farbcodierte Duplexsonographie anschließen, die einen schnellen Überblick über eine pulsierende Raumforderung im Frakturbereich ergibt. Die Kombination einer CTA und DSA erlaubt eine genaue Beurteilung der Ausdehnung eines Pseudoaneurysmas, der umgebenden oder verdrängten Strukturen, eine genaue Lokalisation der Perforationsstelle bzw. exakte Einstufung der Art der Läsion der betroffenen Arterie (und möglicherweise auch assoziierten Vene[nstruktur]) sowie eine Beurteilung der Fraktur und Frakturenden zur Planung einer Rekonstruktion. Das Erkennen von Pseudoaneurysmen nach Schenkelhalsfrakturen und deren Versorgung ist durch regelhafte postoperative Kontrolluntersuchungen schwer möglich, da meist nur Nativröntgenaufnahmen zur Stellungskontrolle der Implantate und Frakturenden durchgeführt werden. In 2 Fallberichten mit Literaturreview der letzten Jahre wurde auf ein Scalloping-Zeichen an der medialen Kortexseite des Oberschenkelknochens als Wirkung des Druckes und der Turbulenzen des Aneurysmas in späteren Phasen der Nativröntgenverlaufskontrolle hingewiesen [8, 9]. Im geschilderten Fallbeispiel fand sich analog dazu eine zirkuläre Hyperostosis. Paraklinische Verlaufsparameter wie ein postoperativer Hämoglobin(Hb)-Abfall konkurrieren mit perioperativen Blutverlusten und sind daher nicht regelhaft zu verwenden.

### Therapie

Als Erstmaßnahme soll das Stufenschema der aktuellen S3-Leitlinie Polytrauma/Schwerverletzten-Behandlung erwähnt werden. Aktive Blutungen der Extremitäten sollen durch folgendes Stufenschema behandelt werden:

1. manuelle Kompression,
2. Kompressionsverband, wenn möglich in Kombination mit einem Hämostyptikum,
3. Tourniquet.

Ein Tourniquet soll dann angewendet werden, wenn eine lebensgefährliche Blutung mit anderen Maßnahmen nicht zeitgerecht gestoppt werden kann [10].

Die offene gefäßchirurgische Rekonstruktion und die endovaskuläre Therapie sollten nicht als konkurrierende Verfahren, sondern als sich ergänzende Methoden zur optimalen Versorgung der Verletzung betrachtet werden. In jedem Fall ist eine patientenspezifische und situationsadaptierte Abwägung des Therapieverfahrens zu wählen. Bei der offenen gefäßchirurgischen Revaskularisierung sollten einige operationstaktische und -technische Erfahrungswerte beachtet werden.

### Fraktur und Gefäßverletzung

Gäbel et al. empfehlen in diesem Falle folgendes Vorgehen: Nach Abstrichentnahme und Wunddébridement mit gründlicher Spülung (ggf. Jet-Lavage) und Desinfektion der Wunde steht nach wie vor die Stabilisierung des Skelettsystems an erster Stelle. Hier hat sich der Fixateur externe als Mittel der Wahl bewährt [11]. Wie oben beschrieben, kann im Einzelfall die Gefäßverletzung zuerst versorgt werden, wenn die Fraktur dem Zugang zum verletzten Gefäß Raum bietet und im Rahmen der Blutstillung sich eine definitive Versorgung gleich anbietet. Gerade bei penetrierenden Halsverletzungen ist allgemein akzeptiert, dass massive Blutung, hypovolämischer Schock, starker Bluthusten und sich ein rasch ausbreitendes Hämatom eine sofortige Indikation zum Eingriff darstellen [12]. Wenn technisch möglich, sollte temporär zunächst ein intravasales Shuntröhrchen verwendet werden, da neben der Reduzierung der Ischämiezeit auch eine Reduktion einer Infektion erfolgt und aufgrund einer Demarkierung avitaler Muskulatur ein adäquates Débridement erleichtert wird [13]. Jedoch steht die Blutungskontrolle an erster Stelle, die je nach Lokalisation der Blutungsquelle und Zugangsweg schnellstmöglich vorgenommen werden muss. Bewährt hat sich einerseits ein Ausklemmen des zuführenden Gefäßes weiter proximal, gegebenenfalls durch einen weiteren Zugang, und die Okklusion des distalen Stumpfendes durch einen Fogarty-Katheter mit 3‑Wege-Hahn [14].

Die einfachste Form der operativen Versorgung besteht in der Direktnaht, auch können Substanzdefekte bis zu einer Länge von 2 cm aufgrund der Dehnungsreserve und nach Mobilisierung der Enden überbrückt werden [15]. Gerade bei kindlichen Verletzungen ist auf die Durchführung von Einzelknopfnähten mit monofilem nichtresorbierbaren Nahtmaterial zu achten [16]. Zu beachten sind in jedem Fall intravasale Thromben, die eine Thrombektomie mit einem Fogarty-Katheter und Spülung mit heparinisierter Kochsalzlösung notwendig machen. Weitere operative Rekonstruktionsmöglichkeiten reichen von einer Patchplastik bis zu einer Bypassrekonstruktion. Aufgrund der generellen Infektionsgefahr offener Verletzungen ist autologem oder xenogenem Patch- oder Bypassmaterial der Vorzug zu geben, wobei das Venenmaterial oberflächlicher Hauptstammvenen wohl am häufigsten Verwendung findet. Dies findet jedoch auch Einschränkungen, einerseits aufgrund

- der Größe des verletzten Gefäßes,
- der Ischämiezeit (gerade bei schwer zu bergenden Unfallopfern vergehen nicht selten Stunden, bis eine operative Versorgung vorgenommen werden kann) und nicht zuletzt
- des Kreislauf- und Gerinnungszustandes des Patienten,

die nach einer kurzen Operationszeit drängen.

In jedem Falle sollte – präoperativ begonnen – eine postoperative kalkulierte Antibiotikatherapie und Heparinisierung je nach Gerinnungsstatus erfolgen.

Unsicherheit kann entstehen, wenn bei kindlichen Frakturen das Gefäß zwar verletzt ist, aber keine relevante Ischämie vorliegt. Dann spricht man von der „pink pulseless hand“. Hier sollte zunächst die Reposition der Fraktur erfolgen. Häufig kommt es danach wieder zu einer Erholung des Pulses. Sollte nach 72 h kein Puls an der distalen betroffenen Extremität vorhanden sein, ist eine weiterführende gefäßchirurgische Diagnostik, ggf. offene Reposition und Gefäßrekonstruktion notwendig [17].

Gerade bei der Rekonstruktion kindlicher Gefäßverletzungen ist die Verwendung eines idealen Gefäßtransplantates wünschenswert. Einigkeit besteht darin, autologem Venenmaterial unbedingtem Vorzug zu geben. Bewährt hat sich hier die Verwendung „umgedrehter“ V.-saphena-magna-Transplantate, die jedoch immer in dem Verdacht einer späteren aneurysmatischen Aufweitung und Dilatation stehen. Cardenau et al. konnten jedoch in ihrer Studie zu Rekonstruktionen mit Saphena-Interponaten an unteren Extremitäten bei Kindern mit einem sehr langen „follow up“ zeigen, dass in ihrer Patientengruppe

- 36 % (5 von 14) unverändert blieben,
- 50 % (7 von 14) eine nichtaneurysmatische Dilatation (und)
- 14 % (2 von 14) eine nichtprogressive aneurysmatische Aufweitung

entwickelten. Ein Transplantat wurde verschlossen und ein Transplantat ging für die Nachsorge verloren. Insgesamt zeigten die Transplantate eine Ausdehnung von 11,2 % nach durchschnittlich 10,7 Jahren postoperativ. Sie schlossen daraus, dass die autogene V. saphena als ein dauerhaftes Transplantat für die arterielle Rekonstruktion bei Kindern mit chronischer Ischämie der unteren Extremitäten angesehen werden kann [18]. In Bezug auf den geschilderten Fall der suprakondylären Humerusfraktur mit Verletzung der A. brachialis favorisierten Lewis et al. die Verwendung der V. basilica aus der Verletzungszone. Zu den Vorteilen zählten sie

- die einzelne Operationswunde auf der weniger auffälligen medialen Seite des Arms,
- eine verkürzte Operationszeit (und)
- die Erhaltung von Spendervenen, die später für die Behandlung von atherosklerotischen Erkrankungen erforderlich sein können [19].

In Bezug auf eigene Behandlungsdaten und damit eruierte Erfahrungen wurden im Universitätsklinikum Magdeburg A. ö. R. von 2013 bis Anfang 2024 9 Kinder mit einer suprakondylären Humerusfraktur und begleitender Gefäßverletzung behandelt. Das Durchschnittsalter betrug 6,78 (Streubreite: 3–10) Jahre. Die operative Therapie erfolgte in 3 Fällen durch Fixierung der Intimastufe und Patchplastik (V. basilica, V. cephalica, V. mediana cubiti). Bei 2 Verletzungen erfolgte die Inspektion und Arteriotomie zur Thrombektomie mit anschließendem Verschluss durch Einzelknopfnähte. Eine Freilegung und Adhäsiolyse war bei 2 Patienten ausreichend, während bei einem Kind eine Resektion mit anschließender End-zu End-Anastomose mit Einzelknopfnähten und bei einem weiteren Kind die Interposition eines Teilstückes der V. basilica erfolgte. So wurden auch in der eigenen Patientenkohorte die Vorteile der Transplantatentnahme in der bereits bestehenden Verletzungszone genutzt. Alle Kinder erhielten vor Entlassung eine Kontrolle mittels Doppler-Ultraschall mit regelrechtem peripheren Flussprofil. Ein weiteres follow-up fand nicht statt.

Andere Erfahrungen setzen auf die Verwendung der V. saphena magna auch bei Kindern und beschreiben im Langzeitverlauf ektatische Umwandlungen. Nicht zuletzt wurde bei Verwendung der V. basilica im Verlauf eine Sklerose beobachtet (persönliche Mitteilung).

### Traumatisches Pseudoaneurysma

Zur Versorgung ergeben sich mehrere Therapiemöglichkeiten. Als sehr unkonventionelle Methode beschrieben Al-Thani et al. die ballongestützte perkutane Thrombininjektion bei einem kritischen COVID-19-Patienten, der ein Pseudoaneurysma der A. subclavia nach Fehlpunktion entwickelte [20], wie auch am Abdomen bei herausfordernden Pseudoaneurysmaätiopathogenesen durchaus erfolgreich gehandhabt [21]. Aufgrund

- der zunehmenden Verfügbarkeit,
- der guten technischen Handhabung (und)
- der hohen technischen Erfolgsquote (als auch)
- des eher limitierten Interventionstraumas

hat sich eine endovaskuläre Versorgung weitgehend durchgesetzt. Weitere Vorteile bieten die Zugangswege über die A. brachialis oder A. femoralis communis durch eine geringe Invasivität sowie deren Kombinationsmöglichkeiten [22]. Bei der Verwendung gecoverter Stents wird in der Regel eine sichere Abdeckung der Perforationsstelle erreicht, eine Kombination mit Coils oder Klebematerialien ist in der Regel nicht nötig. Die offenen operativen Techniken bieten den Vorteil der lokalen Resektion des Pseudoaneurysmas und damit Behandlung der raumfordernden Wirkung, die neben den Stauungssymptomen durch Kompression des venösen Abflusses ebenfalls zu neurologischen Störungen im Bereich des Armes durch Alteration der Plexusstrukturen führen können. Die bei der vorgestellten Patientin aufgetretene Stauungsdermatitis durch Kompression der V. subclavia durch ebensolche Raumforderung war mithin ein Entscheidungskriterium für die offen-gefäßchirurgische Behandlung. Im Vergleich zur endovaskulären Methode sind natürlich die operationsbedingten Komplikationsmöglichkeiten ins Feld zu führen. So sind Verletzungen des Plexus brachialis und der V. subclavia bei operativer Versorgung eines A.-subclavia-Aneurysmas mit einer höheren Morbidität behaftet und unbedingt zu vermeiden.

In der Regel kann ein solcher Arteriendefekt durch eine Direktnaht verschlossen werden, im Akutfall oder bei langstreckigen Verletzungen sind gegebenenfalls ausgedehntere Rekonstruktionen mittels Patchplastiken oder Protheseninterponaten notwendig.

Aufgrund der Seltenheit traumatischer Aneurysmen der A. subclavia erfolgte bei der wissenschaftlichen Beurteilung der Fälle die Recherche der Literatur der vergangenen 5 Jahre mit den Schlüsselwörtern: clavicula – fracture – pseudoaneurysm – subclavian artery in PubMed®. Die Suche ergab 10 Eintrage, wobei es sich bei 8 der Publikationen um „case reports“ und jeweils einer Publikation um ein Review und eine Originalarbeit handelte. Bei einem „case report“ handelte es sich um eine arterielle Thrombose als Komplikation nach Klavikulafraktur, die mit einem karotidobrachialen Bypass behandelt [23] und daher nicht gewertet wurde. Ziel der Originalarbeit war die Bestimmung der anatomischen Beziehung zwischen den Subklaviagefäßen und dem Schlüsselbein, um die sicherste Zone zu bestimmen, die eine Gefäßschädigung während der Operation vermeidet. Dazu wurden die morphometrischen Messungen gesunder Personen anhand 3‑D-rekonstruierter CTA-Bilder analysiert [24].

In den analysierten Fallberichten variierte die Zeit bis zur Diagnose des Pseudoaneurysmas der A. subclavia von unmittelbar bis Monate nach dem Trauma. Während bei den Symptomen Schmerzen und Schwellung im Vordergrund standen, war bei der klinischen Untersuchung in keinem Falle ein Pulsdefizit vorhanden. In der Hälfte der Fälle wurden die Pseudoaneurysmen rein endovaskulär, in 2 Fällen offen und in 2 Fällen im Sinne einer Hybridtechnik behandelt. Die kurze Übersicht bestätigt, dass die endovaskuläre Versorgung der Pseudoaneurysmen der A. subclavia eine sichere Versorgung erlaubt, jedoch auch die offene Versorgung und auch die Kombination mit einem endovaskulären Setting gebräuchlich ist [25–32].

Auch im Bereich eines Profundapseudoaneurysmas nach hüftgelenksnahen Frakturen ist die Embolisation eines meist peripheren Profundaastes eine elegante Methode und deutlich weniger invasiv als die operative Versorgung.

Orapiriyakul et al. [9] analysierten 74 Fälle aus Fallberichten von Pseudoaneurysmen im Zusammenhang mit intertrochantären Fixierungen aus den Jahren 2020 bis 2022. Der häufigste Frakturtyp, der nach AO-Klassifikation auftrat, war eine 31A2-Fraktur (59,5 %). In 9,5 % der Fälle wurden atherosklerotische Gefäße in den Nativröntgenbildern gesehen. Bei intertrochantären Frakturen war in 68,2 % ein kleines trochantärisches Fragment die Ursache für das Pseudoaneurysma. Die meisten Patienten zeigten eine Schwellung (85,1 %) und Schmerzen (70,3 %), nur 1,35 % der Fälle waren asymptomatisch. Sie postulierten prädisponierende Faktoren und Symptome dieser Pseudoaneurysmen, die in Tab. 2 dargestellt werden.

**Tab. 2:** Prädisponierende Faktoren und Symptome von Pseudoaneurysmen im Bereich hüftgelenksnaher Frakturen [11]

	Einzelheiten
Prädisponierende Faktoren	Frakturmerkmale, z. B. Trochanter-minor-Dislokation, Schaftdislokation, lange Spiralfraktur Atherosklerotische Gefäße
Zeichen und Symptome	Raumforderung (ggf. pulsierend), Schwellung und Schmerz sind häufig Zu niedriger Hämatokrit
Ursachen	Frakturstruktur und Dislokation Tiefes Einbringen eines Retraktors Reduktionsmethode, großer Gegenzugstab Stumpfes Trauma Iatrogene Ursachen, z. B. übermäßige Penetration beim Bohren, Drahtcerclage, Manipulation (Innenrotation und Adduktion), hervorstehende Schraube
	Anmerkung: Eine akute Präsentation kann mit der Penetration einer Frakturspitze oder mit einer übermäßigen Penetration beim Bohren zusammenhängen. Eine verzögerte Präsentation kann mit einer hervorstehenden Schraube oder mit atherosklerotischen Gefäßen assoziiert sein.
Diagnostik	Sonographie: Yin-Yang-Zeichen, „to-and-from pattern“ („Hin-und-her-Muster“) Computertomographische Angiographie: kontrastmittelgefüllter Sack mit Bereich geringer Kontrastierung Magnetresonanztomographie: zystische Raumforderung mit Blutkomponenten diverser Aktivierungs- und Prozessierungsgrade
Therapie	Coil-Embolisation Resektion

### Traumatische Aortenverletzung

Bei der Therapie der traumatischen Aortenverletzungen hat sich in den letzten Jahren zunehmend die endovaskuläre Versorgung durchgesetzt. Bedingungen, die ein „thoracic endovascular aortic repair“ (TEVAR) unterstützen, sind:

- multiple schwere Verletzungen,
- keine Beteiligung der Aorta ascendens,
- schwere rechtseitige Thorax- oder Lungenverletzung (Intoleranz einer Intubation),
- geringe Lebenserwartung (und)
- multiple Komorbiditäten [33].

Die häufigste Lokalisation der traumatischen Ruptur ist mit ca. 90 % der (immobile) Aortenisthmus („loco typico“) im proximalen Abschnitt der Aorta thoracica descendens distal der linken A. subclavia mit einem charakteristischen queren Einriss [34].

Die Harborview-Klassifikation nach Heneghan et al. kann bei unterschiedlichen Schweregraden der Verletzungen zur Entscheidungsfindung beitragen (Tab. 3; [35]). Bei der Auswahl der Stentprothese wird ein „oversizing“ von 10–20 % des Aortendurchmessers und eine Landezone von 20 mm empfohlen. Eine Überstentung des A.-subclavia-Abganges mit anschließender Versorgung mit einem Plug wird meist problemlos toleriert, erhöht jedoch das Risiko für eine spinale Ischämie [35]. Neben den bekannten Bypass- und Transpositionsvarianten karotidosubklavial sind auch weitere endovaskuläre Möglichkeiten wie Chimney‑, Snorkel‑, Periskoptechnik oder in-situ-Fenestrierung zur Revaskularisierung der A. subclavia möglich.

**Tab. 3:** Harborview-Klassifikation nach Heneghan et al. [35]

	Beschreibung	Therapie
Minimale Läsion	Keine Konturveränderung der Aorta, Intimaeinriss und/oder Thrombus < 10 mm	Keine Intervention empfohlen, optional nachfolgende Bildgebung
Moderate Läsion	Konturabnormalität der Aorta oder Intimaeinriss > 10 mm	Semielektive Versorgung nach Stabilisierung von Begleitverletzungen
Schwere Läsion	Aktive Extravasation und Hämatom an der linken A. subclavia > 15 mm	Sofortige Versorgung

In Bezug auf eigene Behandlungsdaten und damit eruierten Erfahrungen wurden in der eigenen Patientenklientel seit 2016 12 Fälle mit einer traumatischen Aortenruptur versorgt. In die Auswertung wurden alle konsekutiven Patienten mit einer traumatischen Aortenverletzung jeglichen Unfallmusters aufgenommen. Der Altersdurschnitt lag bei 55 (Streubreite: 24–85, Median: 56) Jahren. Bei 9 Patienten trat die Läsion in der Zone 3 nach Ishimaru, bei 2 Patienten in Zone 4 nach Ishimaru und bei einem Patienten in der Zone 2 nach Ishimaru auf. In den meisten Fällen handelte es sich um polytraumatisierte Patienten. Bei 10 Patienten lag ein Injury Severity Score (ISS) von > 16 vor, bei 6 > 25 und bei 4 Patienten ein Score von ≥ 50. Definitionsgemäß handelt es sich bei einem ISS-Score von ≥ 16 um ein Polytrauma und ab 25 Punkten um ein schweres Polytrauma.

Die Schnitt-Naht-Zeit betrug im Durchschnitt 60,65 (Streubreite: 23–121, Median: 50) min. In 4 Fällen entschied sich der Operateur, die linke A. subclavia zu überstenten. Dies erfolgte 3‑mal bei Verletzungen in Zone 3 nach Ishimaru und 1‑mal in Zone 2 nach Ishimaru. Dies führte bei den Patienten zu keiner unmittelbaren Beeinträchtigung der arteriellen Gefäßversorgung der linken Hand. Bei einem Patienten erfolgte nach 12 Monaten aufgrund beeinträchtigender Belastungsbeschwerden die Anlage eines karotidosubklavialen Bypasses. 3 Patienten verstarben an den Folgen des Polytraumas, bei einem Patienten davon konnte ein axonaler Marklagerschaden und ein intraspinales Hämatom durch das Polytrauma im MRT nachgewiesen werden. Die restlichen 9 Patienten wiesen keine Zeichen einer Paraplegie oder Paraparese durch die Aortenstentung auf. Von den 12 Patienten war ein Patient bereits mit einer aortobiiliacalen Stentprothese bei Aortenaneurysma voroperiert worden. Bei diesem Patienten erfolgte nach 10 Monaten eine distale Stentverlängerung bei distalem Typ-I-Endoleak. In allen Fällen erfolgte die Versorgung mit Gore TAG Conformable® (W. L. Gore & Associates, Inc.), wobei am häufigsten eine Prothese mit einem Durchmesser von 26 mm und einer Länge von 10 cm benutzt wurde. Zusammenfassend wird trotz der statistisch geringen Patientenanzahl in der eigenen Patientenkohorte anhand der Ergebnisse der Nutzen der TEVAR bei traumatischen Aortenrupturen gerade bei schwer polytraumatisierten Patienten durch geringe Operationszeiten, die sicherlich auch von der Erfahrung des Operateurs abhängig sind, und dem geringen Zugangstrauma sichtbar.

Die Diskussion zur Überstentung der A. subclavia links bei einer TEVAR im Falle einer traumatischen Aortenverletzung ist immer wieder Ausgangspunkt für eine Reihe von retrospektiven Analysen und Metaanalysen, da – anatomisch gesehen – der ungehinderte Fluss in die A. subclavia und seinen ersten Ast, der A. vertebralis, als blutversorgendes Gefäß („supply“) der A. spinalis anterior von erheblicher Bedeutung ist. Eine weitere Kollateralquelle der Rückenmarksblutversorgung ist die A. mammaria und deren vordere Interkostaläste. Dieser Weg wird auch durch Überlappung der A. subclavia beeinträchtigt [36]. Chen et al. zeigten in ihrer Metaanalyse von 2019 [37], dass die Revaskularisierung einer abgedeckten linken A. subclavia bei TEVAR mit einem signifikant reduzierten Risiko für

- einen zerebrovaskulären Insult,
- eine Querschnittlähmung (und)
- eine Ischämie der linken oberen Extremität

einhergeht, jedoch nicht mit einem erhöhten Risiko für eine 30-Tage-Mortalität und keine signifikante Schutzwirkung auf eine Querschnittslähmung nach TEVAR hat. Auch die aktuelle retrospektive Studie von Sengör et al. [38] ergab keinen signifikanten Unterschied zwischen den von ihnen untersuchten Gruppen mit und ohne Revaskularisation der linken A. subclavia bei

- 30-Tage-Querschnittslähmung (4,8 % vs. 0,0 %; *p* = 0,449),
- zerebrovaskulären 4‑Jahres-Ereignissen (0,0 % vs. 3,8 %; *p* = 0,998) und
- Ischämie der oberen Extremitäten (9,6 % vs. 0,0 %; *p* = 0,207).

Die Registerstudie von Buth et al. kam jedoch zu dem Schluss, dass eine perioperative Paraplegie oder Paraparese signifikant mit einer Überstentung der linken A. subclavia ohne Revaskularisierung bei verschiedenen Arten thorakaler Aortenpathologien assoziiert war [36]. Innovativen Charakter bei dieser Diskussion hat die in den letzten Jahren eingeführte „Single-branched“-Endoprothese mehrerer Endoprothesenhersteller. Die systematische Metaanalyse von Bontinis et al. [39] konnte aber trotz der Daten

- gepoolter technischer Erfolgs- und 30-Tage-Mortalitätsraten von 94,86 % (95 %-Konfidenzintervall [KI]: 90,95–97,86) und 0,14 % (95 %-KI: 0,00–0,87) sowie
- einer 30-Tage-Schlaganfall- und Rückenmarksverletzungsrate von 0,45 % (95 %-KI: 0,00–1,39) bzw. 0,08 % (95 %-KI: 0,00–0,99)

keine eindeutigen Schlussfolgerungen ziehen. Sie ermutigten zu einer weiteren Forschung durch qualitativ hochwertige randomisierte kontrollierte Studien.

Verglichen mit der eigenen Patientenkohorte, entsprechen die Ergebnisse den Untersuchungen anderer Arbeitsgruppen, wobei die kleine Anzahl an Patienten keiner statistischen Auswertung standhält. Zu bedenken ist die Tatsache, dass bei einem Patienten im Verlauf eine diffuse axonale Schädigung im Marklager sowie ein intraspinales Hämatom nachgewiesen werden konnte. Inwieweit bei einem Polytrauma mit traumatischer Aortenverletzung im Verlauf polytraumabedingte Schäden des Zentralnervensystems bei der Ausbildung einer Paraplegie oder Paraparese mit eine Rolle spielen, ist im Wesentlichen unklar. Auch die Analyse von 11.473 Patienten aus dem nationalen TEVAR-Register der USA durch Scali et al. [40] zeigte für das TEVAR bei traumatischen Aortenverletzungen eine der niedrigsten Inzidenzen von Querschnittlähmung (1,1 %) auf. Sie verwiesen aber auch darauf, dass eine systematische Übersichtarbeit von Lee et al. [41] eine Inzidenz der Querschnittslähmung von 3,3 % aufweist, was zu einer weiteren Beachtung der Problematik in dieser speziellen Patientenklientel führen sollte.

### Stärken

Besonders hervorzuheben ist, dass eindrucksvoll gezeigt wurde, welche Konsequenzen eine Fraktur in der Gefäßumgebung auslösen kann auch im protrahierten Zeitverlauf.

Daraus wird nicht zuletzt sehr deutlich, wie wichtig und teils entscheidend das eher zeitnahe und gewissenhafte, d. h. befundgerechte diagnostische und therapeutische Management ist. Die Repräsentativität diverser Fallkonstellationen und deren klinische Verläufe können das facettenreiche Profil der komplexen anamnese-, symptom-, befund-, diagnose-, therapie- und outcomeassoziierten Aspekte darstellen und im Rahmen der relevanten Manuskriptkategorie einer klinischen Fallserie mit dem Ansatz einer wissenschaftlichen Aufarbeitung widerspiegeln und erörtern als auch einen hohen klinischen Lehrwert (nicht zuletzt auch in der „Auffrischung“), insbesondere auch für den interdisziplinär beteiligten Unfallchirurgen bzw. Orthopäden sowie Kinderchirurgen erzielen.

Adäquates topografisch-anatomisches und pathophysiologisches Grundlagenwissen, vor allem detaillierte fallspezifische Kenntnisse aus zahlreich bestrittenen diagnostischen und therapeutischen Managements und erworbene Erfahrungswerte, erzielt wie in den vorgestellten Kasuistiken, sind Voraussetzung, vergleichbare Fälle diagnosespezifisch und interdisziplinär in angemessener Weise mit hoher prognostischer Erfolgsaussicht im kurzfristigen, aber auch langzeitigen Verlauf zu versorgen.

### Limitation

Als limitierend ist anzuführen, dass es sich „nur“ um eine Fallserie handelt mit definitionsgemäß moderatem Evidenzgrad, um die gewählten Therapiekonzepte in ihrer Qualität und im Outcome zu überprüfen.

## Fazit

Gefäßverletzungen im Zusammenhang mit schweren Traumata und Frakturen können mit unterschiedlichen operativen und endovaskulären/interventionellen Verfahren zunehmend schneller, effizienter und patientenschonender versorgt werden. Trotz der vielfältigen endovaskulären/interventionellen Möglichkeiten sind dennoch die klassischen offenen Revaskularisationsverfahren häufig (noch immer) notwendig und damit teils noch unentbehrlich. Hier gelten nach wie vor die operativen gefäßchirurgischen Grundregeln der Versorgung von Gefäßverletzungen. Insbesondere thorakale Aortenverletzungen bei Hochrasanztraumen, die gewöhnlicherweise eine sehr hohe Mortalität aufweisen, können in gefäßchirurgischen Zentren endovaskulär sicher und zügig behandelt werden. Ein interdisziplinäres Zusammenspiel zwischen Unfallchirurgen, Kinderchirurgen, interventionellen Radiologen und Gefäßchirurgen ist in jedem Fall eine Voraussetzung für eine schnelle und sichere Versorgung schwer traumatisierter Patienten.
